# Advances, Norms, and Perspectives in Product Chemical Footprint Research

**DOI:** 10.3390/ijerph18052728

**Published:** 2021-03-08

**Authors:** Yi Li, Yiman Cheng, Luyao Zhou, Yongliang Yang

**Affiliations:** 1 Fashion Department of International United Faculty between Ningbo University and University of Angers/Faculty of Tourism and Culture, Ningbo University, Ningbo 315201, China; liyi1@nbu.edu.cn; 2 East China Sea Institute/Collaborative Innovation Center of Port Economy, Ningbo University, Ningbo 315211, China; 3 Fashion Institute/Silk and Fashion Culture Research Center of Zhejiang Province, Zhejiang Sci-Tech University, Hangzhou 310018, China; 2018328420001@mails.zstu.edu.cn (Y.C.); 2018328420028@mails.zstu.edu.cn (L.Z.); 4 School of Economics and Management/Ecological Civilization Institute of Zhejiang Province, Zhejiang Sci-Tech University, Hangzhou 310018, China

**Keywords:** chemical footprint, product, calculation, life cycle assessment

## Abstract

The chemical footprint of a product is an important factor for evaluating human toxicity and determining ecotoxic effects caused by chemical pollutants in the entire production cycle and is the premise and effective means to carry out the identification, assessment, and control of chemical, environmental risk. The study reviewed the progress of research on chemical and product chemical footprints. It unified the key issues such as accounting boundaries, data lists, accounting methods, and result evaluation of product chemical footprint calculation. On this basis, we propose methods for evaluating product chemical footprints, providing a normative reference for enterprises and relevant research institutions. The future research is likely to obtain innovative results in the research and application of chemical footprint labels, research on characterization factor calculation methods for chemical substances, construction and standardization of chemical use, and emission database and promotion of a chemical-based guarantee mechanism for environmental management.

## 1. Background

The overuse of chemicals can have serious impacts on human health and ecosystem stability. Chemical pollution has been listed by the United Nations Environment Programm (UNEP) as a globally important environmental issue affecting human survival and development [1].

In terms of ecological health, chemical pollution is considered a major pressure on global biodiversity [2]. Rapid industrialization has contributed to the exponential growth of chemical species. In December 2020, the Chemical Abstracts Service (CAS) included more than 142 million organic and inorganic chemical substances, such as alloys, coordination compounds, minerals, mixtures, polymers, salts, and added 15,000 new substances every day [3]. In 2018, global chemical transactions reached $334.7 billion, and global chemical sales were three times higher than 20 years ago [4]. The number of chemicals has increased, and safety is difficult to ensure; the reality is that chemical pollution from emissions migrates with environmental media, or people consume animals or plants, leading to direct or potential harm from toxic and hazardous substances to the human body. A study conducted by an environmental working group in collaboration with public interest organizations found that the cord blood of 10 babies born in US hospitals in August and September 2004 contained an average of 200 industrial chemicals and pollutants [5]. The Center for Disease Control and Prevention has published data on 352 substances, including dioxins, polychlorinated biphenyls (PCBs), metals, and plastic components that have been detected in the blood and urine of thousands of people [6]. Toxic chemical pollutants can also disrupt the natural stability of ecosystems and trigger pollution problems, such as air pollution, water pollution, and the greenhouse effect, causing serious and irreversible damage. Ambient air pollution was responsible for 7.6% of global deaths in 2016 [7]. Air pollution can affect ecosystems; air pollutants such as Sulphur can cause excessive acidity in lakes and rivers and can damage trees and forest soils [8]. Industrial pollution is pollution directly related to the industry, including toxic chemicals, industrial consumer products, hazardous waste streams, greenhouse gas emissions, which is one of the leading causes of pollution worldwide. Among them, the effectiveness of industrial wastewater treatment has a great impact on the natural environment and human living environment [9]. The production stage of the product involves agriculture, industry, and other fields, and the high level of pollution and hazards of the chemicals they emit make it necessary to study the chemical footprint of products. The research on chemical footprint is mainly carried out on the screening and sequencing of chemicals, concentrate resources on priority management of substances with high emissions, long environmental residence time and high risk to the human body and ecological health, and improving the level of chemical risk control.

In response to the danger of toxic and hazardous chemicals, developed countries regulate chemicals by enacting updated regulations, among which the European Union (EU) implemented “the Registration, Evaluation, Authorization, and Restriction of Chemicals (REACH)” regulation, which implements a comprehensive registration, evaluation, authorization and restriction system for chemicals in the EU [10]; the US enacted “the Toxic Substances Control Act (TSCA)” to ban/restrict the production and sale of high-risk chemicals to control chemical risks at source; Japan amended “Chemical Substance Control Law (CSCL)” to regulate the import declaration of industrial chemicals, and Korea enacted the K-REACH regulation to regulate new chemical substances and designated existing chemical substances [11]. In addition to the issuance of regulatory regulations, horizontal cooperation between the government, chemical suppliers, manufacturers, and other organizations has taken place accordingly, including the Zero Discharge of Hazardous Chemicals (ZDHC), which unites more than 160 contributors from the fashion and footwear industries to work toward zero discharge of hazardous chemicals [12]; in December 2020, the China National Textile and apparel council (CNTAC) established the “China Textile and Apparel Industry Whole Life Cycle Assessment(LCA) Working Group (CNTAC-LCA),” which includes private companies and academic institutions, to further promote energy saving and emission reduction of enterprises and products through the measurement and analysis of product carbon footprint, chemical footprint and other environmental indicators through the LCA evaluation system and tools for textile products [13]. However, these policies are only regulatory instruments. They lack quantitative methods and uniform management norms for environmental load evaluation of chemical use and emissions throughout the life cycle of industrial production of products.

As an environmental footprint, the chemical footprint (ChF), which inherits the characteristics of the footprint approach [14], can be used in evaluating the impact of chemical contamination from human activities on environmental sustainability, facilitates the identification of situations that could enable us to prevent “chemical overshoot” beyond the Earth’s safe operating space [15], and is one of the most commonly used quantitative methods for assessing toxicological pressure on human and ecological chemicals. It has been applied at different levels, particularly at the product [16,17,18,19,20,21,22,23], industry [24], enterprise [25], and country [26,27,28] levels. This paper reviews the progress of research on chemical footprints worldwide, finds that there is a lack of guiding norms and processes for product chemical footprint calculation and evaluation. The contribution of this study is to unify the key issues such as accounting boundary, research method, and evaluation of product chemical footprint, so as to strengthen the normality, operability, and process of research and improve the risk identification, assessment, and management of industrial chemicals in the product production process.

## 2. Concept of Product Chemical Footprint

**Figure 1:**
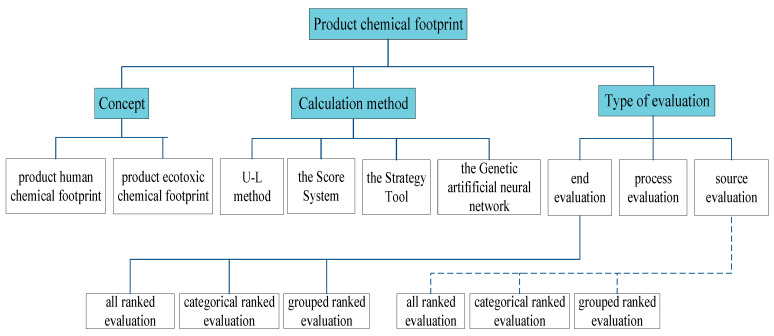
Overview of the conceptual classification of product chemical footprints based on toxic pressure.

With the massive use of natural resources and increasing emissions levels in water, soil, and air, environmental problems are becoming increasingly severe. To evaluate environmental load caused by human activities, many researchers have exerted considerable effort to study carbon, water, and environmental footprints. However, most of them have not considered the sources of relevant substances that impact ecosystems and human health, that is, the sources of chemical pollutants [29]. The chemical footprint, as a footprint-type indicator, facilitates and standardizes the study of the impact of thousands of chemicals on environmental sustainability. The Figure 1 shows the outline of the product chemical footprint concept in terms of concept, calculation method, and type of evaluation. In the major literature databases such as Web of Science and Elsevier, the literature was searched by the keywords chemical footprint, ecotoxicity, toxicity, life cycle assessment, USETOX, characterization factor (CF), life cycle impact assessment (LCIA), etc., and the product categories were selected from them (Table 1).

The concept of chemical footprint has not been elucidated. From the perspective of toxic stress, that is, the carrying capacity of ecosystems, the chemical footprint characterizes the impact of a chemical release on ecosystems and human health over time and in a given spatial volume [16,19,30,31]. From the perspective of environmental space occupancy, the chemical footprint is the quantification and evaluation of resources occupied by diluting chemical emissions to concentrations that are not harmful to the environment [15,29,32,33]. From a qualitative perspective, the chemical footprint refers to quantify the number of hazardous components in a product’s life cycle and the potential risks it poses to humans and ecosystems [34].

By contrast, the toxicity stress is effective in evaluating the effects of pollutants on the human body and ecotoxicity, that is, the possible consequences of chemical pollution on the ecological environment and human body. It is an indicator of impact. Chemical footprints characterized based on space occupancy and mass expressions are compared by quantitative analysis. The quantity-based indicators of chemical pollutants are widely used mainly because data for comparing decision-making units are easily available [26]. However, mass-based indicators should not be considered a substitute for (ecological) toxicity impact potential, as they do not consider the fate, exposure, and impact of substances; moreover, in the driver-pressure-state-impact-response framework, the mass-based indicators of chemical pollution are stress indicators [28]. Therefore, Toxicity stress is suitable for calculating and evaluating complex and diverse chemical footprints.

Depending on the subject, the product chemical footprint is divided into two characteristic forms of products: human toxic chemical and product ecotoxic chemical footprints. Product human toxic chemical footprint (ChF_hum_pro_) refers to the number of human diseases caused by the discharge of chemical pollutants per unit of the product during the production process (cases·kg^-1^/cases·m^-1^/cases·piece^-1^). Product ecotoxic chemical footprint (ChF_eco_pro_) refers to the proportion of the potential impact of species within a certain time and the volume caused by the discharge of chemical pollutants into an environmental medium per unit of product ([PAF]m^3^·day·kg^-1^/[PAF]m^3^·day·m^-1^/[PAF]m^3^·day·piece^-1^). Roos et al. [19] were the first to consider the chemical footprint of textile products, to explore the applicability of the LCA to the textile industry and the impact of chemicals on the environment. Since then, Qian J.H. et al. [16], Berthoud et al. [17] and Li Y. et al. [18] have calculated and evaluated the chemical footprints of products with USEtox and LCA (see Table 1 and Figure 1).

Three methods are used in characterizing product chemical footprint, namely, the score system, the chemical footprint calculation method based on USEtox and LCA (U-L method), the strategy tool, and the genetic artificial neural network (ANN). Among them, launched in 2005 by UNEP and the Society of Environmental Toxicology and Chemistry (SETAC), the USEtox model organizes an international team of LCA experts to make extensive comparisons of existing LCA models [35], which contain a database of more than 3000 chemical substances and contains manual entry interfaces for new substances. USEtox provides a concise and transparent tool for human health and ecosystem assessment and is based on a reference database, which is used in calculating CFs for thousands of substances and forms the basis of the UNEP-SETAC recommendations of the Life Cycle Initiative on the characterization of toxic effects in life cycle assessment [36]. The scoring system developed in the 1990s by the federation of Danish textile and clothing [37] is a semi-quantitative multi-criteria analysis method [38] for describing the properties of chemical substances and determining the scale of their use in the production process [39]. A score of 1–4 was assigned to each substance according to the following criteria: A-mass of weekly discharge, B-biodegradability, C-bioconcentration factor, and D-toxicity. The scores for the four criteria are multiplied. The lowest value of 1 indicates the best environmental performance, and the highest value of 256 indicates the worst environmental performance. A value of 4 is assigned to a substance with the highest score but has no information. The strategy tool was developed by Askham et al. [40] to make strategic decisions about product development. It assesses the chemical composition of a product in a simplified way based on information available in Safety Data Sheets (SDS) [39]. Ping Hou et al. developed a neural network model with an architecture optimized by a genetic algorithm to efficiently predict the ecotoxicity of chemicals (HC50 values in USEtox) [41].

Roos and Peters presented and compared three different methods for evaluating product toxicity in an LCA environment, using the wet treatment process of a cotton t-shirt as an example. These methods have advantages and disadvantages. The advantage of USEtox is that it is a quantitative evaluation method for simulating and assessing the toxicity of actual substances emitted to different compartments [39]. It is useful in determining where and how different assumptions, scenarios, and decision choices give rise to differences among model outputs [42]. The drawback of the USEtox model is that it does not include a high level of spatial resolution and metal database coverage [43]. The advantage of the scoring system is its simplicity, and the time frame for its implementation and use ranges from days to weeks; the disadvantage of the scoring system is that it is limited in scope, as it only deals with the release of environmentally harmful substances to water. The strategy tool has the advantage of being simple and including exposures in the work environment; it has the disadvantage of not being universally applicable throughout the life cycle [39]. The scoring system and strategy tool are qualitative calculation methods with some subjective limitations. Neural network models can quickly predict the ecotoxicity of chemicals and fill in data gaps (HC50), but the disadvantage is that extrapolated HC50 has uncertainty, and model results may not be directly applicable to risk assessment [41].

The key to solving environmental pollution problems is to identify hazards and trace the sources of priority pollutants [26]. The types of methods for evaluating the chemical footprint of a product can be divided into three categories, namely, process evaluation [16,17,18], end evaluation [19,20,21,22], and source evaluation [23]. Taking the study of Roos [19], Van Hoof [20], García [21], and Elorri Igos [22] (Table 1) as an example, end evaluation refers to the evaluation of end pollutants emitted after product production is completed and the harmful effects of the disposal stage to humans and ecosystems. End evaluation can be divided into categorical all ranked evaluation, categorical ranked evaluation, and grouped ranked evaluation. Categorical all ranking refers to the sorting of all pollutants, to arrive at the most toxic impact of the product; categorical ranking refers to the inductive classification of pollutants according to their different chemical composition and structure, such as heavy metals and fluorine-containing categories, which are sorted according to the toxicity size of each category of pollutants; grouped ranking refers to the re-ordering of pollutants within each category, such as copper, cobalt, and chromium in the heavy metals category. End evaluation can be sorted and enables the targeted control of pollutants, but the essence is the passive control of results, end-treatment is expensive and the amount of pollution is too large to solve the root of the pollution problem.

Chemical governance should shift from pollution management to risk prevention [44]. As a risk prevention tool, process evaluation refers to the evaluation of toxic stress caused by chemicals used in the product-manufacturing process. Product life cycle assessment is essentially process evaluation, including raw material production, product processing, marketing, and end treatment. Qian J.H. et al. [16], Berthoud et al. [17], and Li Y. et al. [18] evaluated the chemical footprint of the product manufacturing process based on the LCA theory and the type of evaluation is process evaluation. Process evaluation has the advantage of reducing the generation of textile chemical pollutants and reducing the high costs incurred during end control [45]. However, this method firstly requires a complete inventory of source inputs, production conversion, and end emissions of chemicals, which requires higher quality of data, more difficult data collection, higher time and economic costs.

The design/development phase is often excluded in life cycle assessment because it has little impact on environmental loads. However, decisions made during the design/development phase have a significant environmental impact on other life cycle phases [46]. Source evaluation can be divided into two categories, namely the design and development phases. The first type of source control involves the legal and regulatory restriction of the use of highly hazardous chemicals, that is, the Restricted Chemicals List, similar to the REACH regulation, the ZDHC Manufacturing Restricted Substances List. Another source control requires the end pollutant combined with the process chain to reverse the source input substance that caused the specific pollutant introducing, which targets non-toxic or less harmful chemicals. The reason is that after the chemicals are put into production, many discharged chemicals are converted into other chemicals through biological and physical-chemical processes [47], and the degradation products are bio-cumulative and toxic. The other reason is excessive use, which results in toxicity after end-of-discharge. Most studies focused on process and end evaluation for the following reasons: the susceptibility of input chemicals to chemical reactions during the production process, the complexity of the process chain, the variety of chemical uses and properties, the absence of characterization factors in calculation databases, and the lack of sound pollutant input, transfer, and output inventories by companies. Li Y et al. [23] accounted for the chemical footprint of textile and apparel products to identify chemical categories with large toxic effects, including the initial inputs of chemical raw materials and final emissions of chemical pollutants. The ChF results of chemical materials show that antifoaming agents in the warp dyeing phase are highly toxic to humans. In the weft bleaching phase, peroxide stabilizer is highly toxic to humans and wetting/penetrating and sequestering agents are highly toxic to the ecological environment.

In contrast to water, soil, and air medium management, as a chemical substance management, the production process of product chemicals is complicated. The amount and variety of chemical pollutants are huge, chemical management relies only on end evaluation and process evaluation defects obviously. The study advocates source control because this approach enables the active control and prevention of risks, thereby potentially reducing the level of cumulative emission in the production process to a zero-emission level. This approach also mitigates pollution in the production process and allows manufacturers to establish an environmentally friendly image, further maintains ecological stability, and promotes human health. The data required for calculating the chemical footprint, including the characterization factors in the USEtox database and the estimated factors based on USEtox, chemical types, and input and output quantities are derived from experimental or agency data, similar to The European Pollutant Release and Transfer Register (E-PRTR) and the US Environmental Protection Agency’s Toxics Release Inventory. Through the study and analysis of the existing literature on the chemical footprints of products, the following norms should be formed for the calculation and evaluation of the chemical footprint of products.

## 3. Norms for Product Chemical Footprint Calculation and Evaluation

Based on the existing research literature on chemical footprint and the whole life cycle production process of different products, this paper constructs a process framework for calculating and evaluating the chemical footprint of products, i.e., “definition of accounting boundary, construction of accounting data list, construction of a calculation model and evaluation of calculation results.” The aim is to apply this process to studying human toxicity and ecotoxicity effects of various products. In the study, a flow chart of product production and manufacturing is established, the boundaries of product chemical footprint accounting are delineated, a data list is constructed based on the mechanism of chemical input conversion, the chemical footprints of products located in the same region, and those located in different regions in the production area are proposed and a comprehensive evaluation method for human toxicity and ecotoxicity is constructed.

### 3.1. Determination of Accounting Boundary

The LCA is used in assessing the potential environmental impacts and resources used throughout the life cycle of a product, from the raw material sourcing, production, and use stages to waste management [48]. LCA is the main method for the environmental impact assessment of products [17]. The LCA technical framework can be divided into goal definition and scoping, inventory analysis, impact assessment, and improvement assessment [49]. System boundaries describing the study and defining functional units are included in goal definition and scoping, and temporal and geographical constraints can be called system boundaries [50]. The temporal boundary is the period between the beginning and end of the life cycle of a product. The beginning should be traced back to raw materials in nature, and the end should be the output of waste to nature. The spatial boundary includes the various material inputs and outputs in a product being accounted for within the temporal boundary.

**Figure 2:**
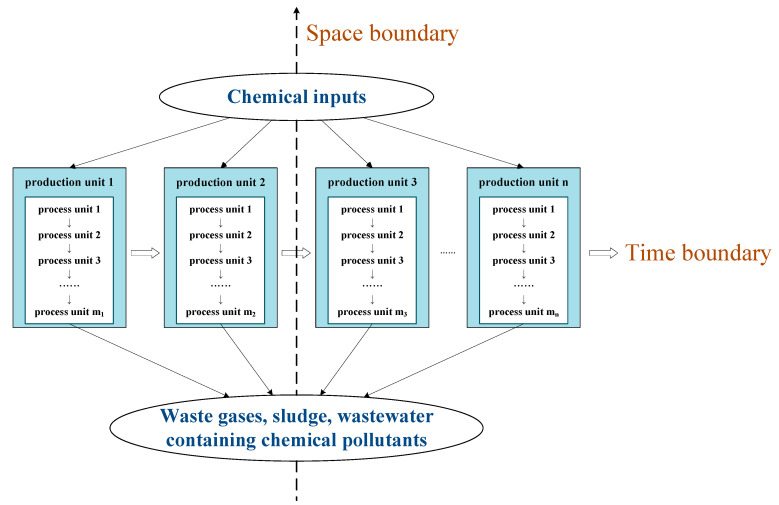
Modular model for hierarchical product chemical footprint calculation.

In the study of a product’s chemical footprint, understanding the production process, that is, the “production system” of the product, is necessary. According to the product flow diagram (Figure 2), the first-level module of the production chain is the main process of product production. Based on the complexity of product function and structure, the process chain can be subdivided under the module contained in the first level of the production chain, thus forming the second level of the process chain. For example, the first module of the production chain of textile and apparel is divided into fiber production, spinning, weaving, dyeing and finishing, finished product processing, and sales. Fiber production, in turn, involves pulp, dissolution, filtration, (defoaming, spinning), washing, bleaching, oiling, drying, spinning, and weaving. Environmental exchange is usually assumed to be linearly related to a product stream of the unit process [46]. Corresponding the process chain to a temporal boundary, the inputs are the chemicals used in the product production process, and the outputs are the exhaust gases, sludge, and wastewater containing chemical pollutants. These outputs are produced during and after the production process. Energy production, fuel use, and the amount of chemicals reacted are not included. The determination of accounting boundaries is a prerequisite and primary specification for carrying out chemical footprint calculation, and also ensures comparability of accounting results; modularity facilitates the construction, collection, and proofreading of data inventories. Figure 2 shows modular model for hierarchical product chemical footprint calculation.

### 3.2. Inventory of Accounting Data

**Figure 3:**
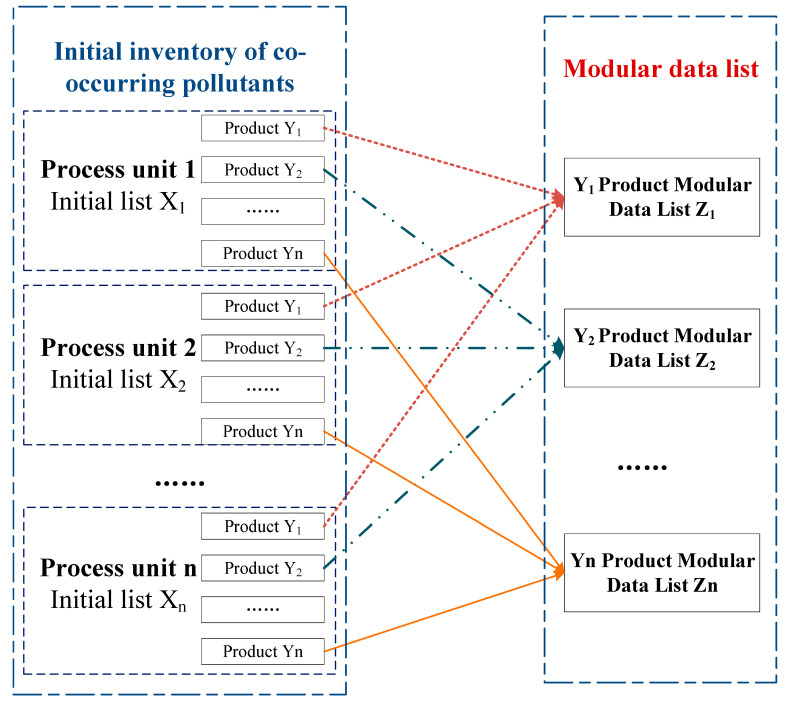
Product modular co-pollutant splitting and recombination principles.

LCA is usually limited to products generated or used in a specific region at a specific time, but three scenarios are possible when a process is shared by multiple product systems: multi-output, multi-input, and open-loop recycling [50]. The solution to the above multiple allocation problem combines two approaches. The first is the conservation of material flow, using the mechanism of chemical input and transformation, that is, the input of m n-mass chemical yields x y-mass pollutants; the second is to refine the production process and split and reorganize the initial list of symbiotic contaminants. As shown in Figure 3, a modular data list for an individual product was built, which is a compilation of relevant input and output chemicals required for the product system. The construction of the data list is the basis for product chemical footprint calculation and chemical environmental risk prevention and management. In the construction of a data list for multi-product production, the unification of functional units requires attention. The quantities of each product, pollutant, and resource must be measured in the same way as in each unit process, and the terminology used to represent flows and other environmental exchanges should be consistent [46].

### 3.3. Building a Calculation Model

#### 3.3.1. Calculation Methods for Chemical Footprinting Based on the USEtox Model and LCA Theory

The USEtox model is a scientific consensus model developed by the UNEP-Society of Environmental Toxicology and Chemistry based on comparative studies of similar models for characterising the effects of chemical releases on human toxicity and ecotoxicity in LCA [36,43]. Sala et al. [29] were the first to propose the use of the USEtox model in the development of chemical footprints, combining a lifecycle-based approach with methods developed in other contexts, such as risk assessment and sustainability science. The CF is divided into human toxicity characterization factor (CF_hum_) and ecotoxicity characterization factor (CF_eco_). CF_hum_ is divided into mid-point and end-point levels, the midpoint level is expressed as the number of cases of diseases caused by the emission of chemical pollutants unit mass (cases·kg^−1^_emitted_). End-point levels are indicated by disability-adjusted life year (DALY) (DALY·kg^−1^_emitted_). CF_eco_ is divided into mid-point and end-point levels. The mid-point level represents chemical substances released to an environmental medium (per unit mass) and contributes to the potential impacts of species at a given time and volume ([PAF]m^3^·day·kg^−1^_emitted_). The end-point level is indicated by the proportion of potential extinction of species ([PDF]m^3^·day·kg^−1^_emitted_). The CFs can be obtained from the latest version of the USEtox model. According to Ralph K. Rosenbaum et al. [36], the formula is as follows.

CF=FF⋅XF⋅EF

*FF* is the fate factor, *XF* is the exposure factor, and *EF* is the effect factor. Bjørn et al. [51] introduced a chemical footprinting approach that expresses the ecotoxic effects of anthropogenic chemical emissions to prevent the damage to freshwater ecosystems. The human and ecological footprints of a chemical are obtained by weighted summation and are expressed as impact scores through the following formula:

$$IS=\sum_{i}\sum_{x}CF_{x,i}\times m_{x,i}$$

*IS* is the impact score for the chemical, *CF_x,i_* is the CF for releasing chemical *x* to an environmental medium *i,* and *m_x,i_* is the mass of chemical *x* released to environmental medium *i*.

#### 3.3.2. Methodology for Calculating the Chemical Footprints of Products in the Same Region

**Figure 4:**
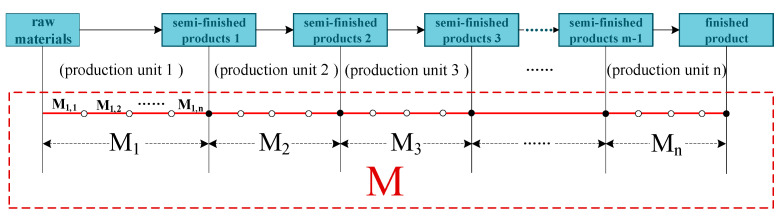
Product life-cycle stages.

When the production of products is concentrated in one area, the process chain of product production is divided into individual production units M1, M2, M3, L, Mn. The processes involved in the production units are accounted for separately, and the total product chemical footprint is the sum of the chemical footprints of each production unit (as shown in Figure 4).

Calculating Formula (1) is as follows.

(1) $$ChF_{M}=\sum_{y=1}^{n}\sum_{x=1}^{m}ChF_{M_{x,y}}=\left\{\begin{array}{l} \sum_{y=1}^{n}ChF_{M_{1,y}}\left(x\leq 1\right)\\ ChF_{M_{1}}+\sum_{y=1}^{n}ChF_{M_{2,y}}\left(1<x\leq 2\right)\\ ChF_{M_{1}}+ChF_{M_{2}}+\sum_{y=1}^{n}ChF_{M_{3,y}}\left(2<x\leq 3\right)\\ \cdots \cdots \\ ChF_{M_{1}}+ChF_{M_{2}}+\cdots \cdots +\sum_{y=1}^{n}ChF_{M_{m,y}}\left(m-1<x\leq m\right) \end{array}\right.$$

where *ChF_M_* is the total chemical footprint of the product, *x* is the product category of each production unit after completion, 1 ≤ *x* ≤ *m*, the production unit includes multiple process units, *y* is the number of process units, *y* ≥ 1.

(2) $$ChF_{pro}=\frac{ChF_{M}}{Q_{M}}=\frac{f\cdot {\left(\sum_{j=1}^{8}\sum_{i=1}^{n}CF_{i,j}\cdot E_{i,j}\right)}_{M}}{Q_{M}}$$

*ChF_pro_* is the chemical footprint of per functional unit product, *Q_M_* is the total mass of the product, *CF_i,j_* is the CF of chemical *i* emitted to environmental media *j*, *E_i,j_* is the quality of chemical *i* emitted to environmental media *j* within the system boundary, and *f* is the conversion correction factor between the characteristic factor of the USEtox model and the characteristic factor of the chemical footprint, with a value of 290 and without dimension. The formula for the chemical footprint of human and ecological products is similar to Formulas (2)–(5). The formula for calculating the chemical footprint of human products (3) and the formula for calculating the chemical footprint of ecological products (4) are in the following order:

(3) $$ChF_{hum\_pro}=\frac{ChF_{M}}{Q_{M}}=\frac{f\cdot {\left(\sum_{j=1}^{8}\sum_{i=1}^{n}CF_{hum_{i,j}}\cdot E_{i,j}\right)}_{M}}{Q_{M}}$$

(4) $$ChF_{eco\_pro}=\frac{ChF_{M}}{Q_{M}}=\frac{f\cdot {\left(\sum_{j=1}^{8}\sum_{i=1}^{n}CF_{eco_{i,j}}\cdot E_{i,j}\right)}_{M}}{Q_{M}}$$

#### 3.3.3. Methodology for Calculating the Chemical Footprint of Products in Different Regions

When products are produced in different regions, the effect of the receiving environment on toxicity varies, depending on the transport and exposure of contaminants in different environmental media [25]. Wang L.L. [52] converted a product chemical footprint into a regional chemical footprint by using the natural background chemical footprint and the regional toxicity stress index (RTSI), considering the differences among the natural environmental backgrounds of chemical pollutants in different regions. RTSI is used to express the degree of the environmental impact of industrial production activity in different regions in a particular year [52], which can be used in calculating and evaluating the chemical footprint of a product produced and processed in different regions.

First, the data lookup obtains the underlying data sources of selected regions and obtains s years of chemical pollutant emissions from designated production areas. Natural background chemical footprint *ChF*_B_ in the *s*-th year can be obtained as follows:

(5) $$ChF_{B}=\overset{l}{\underset{i=1}{\sum }}\left[\overset{j}{\underset{n=0}{\sum }}\left(1-n\times kdeg_{w^{i}}\right)\times \overset{k}{\underset{x=1}{\sum }}\overset{2}{\underset{p=1}{\sum }}\left(m_{pxi}\times f\times CF_{i}\right)\right]$$

As in Formula (6), the annual degradation rate $kdeg_{w^{i}}$ is.

(6) $$kdeg_{w^{i}}=365\times 24\times 60\times 60\times kdeg_{w}$$

where *i* is the *i*-th pollutant, with a total number of l; *n* is the *n*-th year before that year, the total number of years is j; *x* is the *x*-th environmental medium, the total number is k; *p* is the *p*-th pollutant, 1 represents industrial effluent, 2 represents domestic effluent; $kdeg_{w^{i}}$ represents the degradation rate of the *i*-th pollutant emitted by the product in y^-1^; *m_pxi_* represents the amount of pollutant *i* emitted by *p* categories of pollutants in year n; *CF_i_* is the toxicity CF of the *i*-th pollutant; and $kdeg_{w}$ is the rate of degradation of matter *i* per second at a temperature of 25 °C, at standard atmospheric pressure.

Based on the results of Formula (5), the Regional Toxic Stress Index for product *c* in the *s*-th year of production is calculated as follows:

(7) $$RTSI_{S}=\frac{ChF_{C}}{ChF_{C}+ChF_{B}}=\frac{1}{\frac{ChF_{B}}{ChF_{C}}+1}$$

*ChFc* is the chemical footprint of product *c*; *ChF_B_* is the natural background chemical footprint in *s*-th year. When the chemical footprint of product *c* remains the same, the larger the *ChF_B_*, the lower the environmental impact industrial activities have on particular industrial production activity in a designated area.

As in Formula (8), calculate the product chemical footprint *ChF_q_* under regional stress.

(8) $$ChF_{q}=ChF_{C}\times RTSI_{S}$$

**Figure 5:**
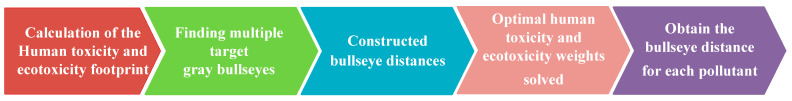
Integrated human toxicity and ecotoxicity evaluation steps.

Figure 5 shows integrated human toxicity and ecotoxicity evaluation steps.

#### 3.3.4. Integrated Evaluation of Human Toxicity and Ecotoxicity

Human toxicity and ecotoxicity footprints are the two types of units of different nature and cannot be evaluated comprehensively for specific impacts [31], and they lack comparability. In order to be able to comprehensively evaluate the toxicity impact of product chemicals and enhance the comparability of chemical footprint calculation results for different products, different processes, and different regions, the human toxicity and ecotoxicity can be comprehensively evaluated based on the target theory, and the grey extremity transformation method can be used within the accounting boundary for the identification of the multiple target grey bullseye, and the bullseye distance can be obtained through the optimum value of the human toxicity and ecotoxicity footprint weights. The steps are as follows: (As shown in Figure 5)

1. The human toxicity and ecotoxicity footprint are calculated (9). Toxic impact on the environment and humans decreases with the combined value of human toxicity and ecotoxicity. The grey extremity transformation formula is

(9) $$r_{i}^{\left(k\right)}=\frac{max\left\{u^{\left(k\right)}\right\}-u_{i}^{\left(k\right)}}{max\left\{u^{\left(k\right)}\right\}-min\left\{u^{\left(k\right)}\right\}}$$

where 0 ≤ *r_i_^(k)^* ≤ 1, *k* is the human toxicity and ecotoxicity evaluation indicator, *k* = 1, 2; *u_i_^(k)^* is the size of human toxicity (*k*_1_) and ecotoxicity (*k*_2_) of chemical pollutant *i*, *i* = 1, 2, …, m; min{*u^(k)^*} and max{*u^(k)^*} are the minimum and maximum toxicity values under human toxicity or ecotoxicity index, respectively.

2. Multiple target grey bullseyes are determined.

(10) $$r=\left(max\left\{r^{\left(1\right)}\right\},max\left\{r^{\left(2\right)}\right\}\right)$$

is a point in a 2-dimensional coordinate system.

3. Bullseye distances *d_i_* are constructed, that is, the distances of human toxicity and ecotoxicity from multi-target grey targets in the coordinate system.

(11) $$d_{i}=\sum_{k=1}^{2}\omega_{k}\left|r_{i}^{\left(k\right)}-max\left\{r^{\left(k\right)}\right\}\right|$$

where *ω_k_* is the weight of the human toxicity and ecotoxicity evaluation indicator *k*.

4. The integrated bullseye distance is minimized by using a single-objective optimization model as an objective function for solving optimal human toxicity and ecotoxicity weights.

(12) $$min\varepsilon_{i}^{2}=\sum_{i=1}^{m}\sum_{k=1}^{2}\omega_{k}^{2}{\left[d_{i}^{\left(k\right)}\right]}^{2}$$

(13) $$s.t.\overset{2}{\underset{k=1}{\sum }}\omega_{k}=1,0\leq \omega_{k}\leq 1$$

where $d_{i}^{\left(k\right)}=\left|r_{i}^{\left(k\right)}-max\left\{r^{\left(k\right)}\right\}\right|$. The solution is

(14) $$\omega_{k}^{*}=\frac{1}{\sum_{k=1}^{2}\frac{1}{\sum_{i=1}^{m}{\left[d_{i}^{\left(k\right)}\right]}^{2}}}\cdot \frac{1}{\sum_{i=1}^{m}{\left[d_{i}^{\left(k\right)}\right]}^{2}}$$

5. The obtained weights for each indicator are substituted into Formula (11) to calculate bullseye distance for each pollutant.

(15) $$d_{i}^{*}=\sum_{k=1}^{2}\omega_{k}^{*}\left|r_{i}^{\left(k\right)}-max\left\{r^{\left(k\right)}\right\}\right|$$

The size of the human and ecotoxic chemical footprint of each contaminant can be ranked according to the size of the bullseye distance *d_i_^∗^*, and chemical pressure on the environment decreases with *d_i_^∗^*.

### 3.4. Evaluation of Calculation Results

By analyzing the above-mentioned product chemical footprint calculation results and evaluating the impact of human toxicity and ecotoxicity, we can find the severely polluting chemicals or process stages for enterprises, provide specific countermeasures for enterprises and enable them to improve their chemical footprint and provide reasonable suggestions for the formulation of green development strategies. The evaluation method is categorized according to different perspectives, including the life cycle stage (source, middle, and end) of product production [53], the level of the product (organization, region, and country) [54], calculation methods (the score system, the chemical footprint calculation method based on USEtox and LCA and the strategy tool) [39] and calculation models (USEtox, COEMEDE) [55]. The selection of an evaluation perspective is based on the study’s purpose, which is mainly based on the chemical footprint of products in the whole life cycle. The type of evaluation is in the whole life cycle stage, the study mainly exploring severely polluting chemicals, so the evaluation includes priority control pollutants, source, and process chain end evaluation. Summarising the existing literature, the evaluation of the chemical footprint can be carried out from four aspects.

1. Evaluation of priority control pollutants. The evaluation of priority control pollutants refers to measuring the impact of pollutants on human beings and ecology. These pollutants are produced by chemicals used in the product processing process. When pollutants exert toxic effects, enterprises can replace chemicals with those providing equal benefits and has lower toxicity or reduce the number of chemicals injected to reduce toxic effects on humans and the ecology. However, due to the different toxicogenic modes of chemical substances and the joint action between substances, the hazards are difficult to predict, and the technical difficulties of end management and government regulatory pressure are great.
2. Source evaluation of priority control pollutants. Priority control pollutant source evaluation means tracing the sources of pollutants according to the toxicity of emitted chemicals and determining chemical inputs that are responsible for the toxicity of these pollutants. Based on the results of human toxicity and ecotoxic chemical footprint accounting, the sources of pollutants can be traced by combining the toxic contributions of pollutants in various raw materials of products. Chemical environmental management should shift from traditional end-of-pollution control to source risk prevention and control. Controlling chemicals at the source is an important prerequisite for transforming the environmental management of chemicals from hazard management to risk management, and helps promote the transfer of the entire chemical management regulatory system to the enterprises themselves.
3. Evaluation of priority control process chains. The evaluation of the priority control process chain refers to evaluating the chemical footprints of products in each process chain in the production and processing of products. It is divided into the priority control process evaluation and priority control process chain evaluation. Enterprises may replace machines or change processes to reduce the chemical footprints of products.
4. Evaluation of the chemical footprints of products in different regions. The evaluation of the chemical footprints of products in different regions refers to measuring the degree of environmental impact caused by the production and processing of products in different regions. Different regions have different levels of chemical pollution and different production conditions and product characteristics. Thus, the location of a production region affects the quantitative size of the chemical footprint of a product. Calculating the chemical footprint of products in different regions and selecting regions with low ecotoxicity stress can facilitate enterprises or industries to choose suitable production regions.

## 4. Prospect

The chemical footprint of a product is useful in measuring the toxic effects of chemical pollutants on human beings and ecology during the production process of products for the reduction of environmental pollution and the protection of public health. However, it has many issues, which require further discussion and must be solved by many research institutes. Innovative results are possible in four areas.

1. Research and application of chemical footprint labels. Lack of transparency and metrics for address complex chemicals management in the supply chain creates a significant barrier for many consumer brands [34]. Like the carbon footprint label and environmental footprint label, the chemical footprint produced by-products during their life cycle is presented in the form of a quantitative index on the product label. The chemical footprint label has two separate components: a detailed and precise ranking of the chemical footprint data contained in the product and the way this information is transmitted to users. By bringing together manufacturers, retailers, and users (consumers, governments, and foodservice providers), every member of the supply chain needs to know chemical footprint information [56]. Chemical footprint labelling helps in raising awareness of the extent of chemical contamination among participants and facilitates the creation of an environmentally friendly image for suppliers while furthering the development of the supply chain.
2. Research on the calculation method of chemical substance CF. The CF is the main table parameter for chemical footprint accounting. The database embedded in the USEtox model contains more than 3000 chemical substances and their CFs. However, the rate of updating the CFs of chemical substances is much slower than research and development and production of innovative chemical substances. USEtox is developed for organic matter, but some substances cannot be represented, including particulate matter, nitrogen oxides, sulphur oxides, chlorides, fluorides, and cyanides, some of which are highly relevant from an (ecological) toxicological point of view (e.g., cyanide) [28]. The absence of CFs for these chemical substances impedes the evaluation of the toxicity of chemicals to humans and ecosystems. The USEtox manual [57] provides clear guidance on the use of estimated data in calculating fate factors. The handbook opens up the use of estimated data but provides no further guidance on ecotoxicological influences and explicitly states that experimental data should be used in calculating human health influences [55]. The estimation of CFs lacks accuracy due to the complexity of the chemical structure of substances. Thus, developing methods for determining missing factors is necessary.
3. Construction of a database on chemical use and release and study of regulations. The use of chemicals covers the whole life cycle of product production, in which each link produces a chemical footprint. The use of process control in long industrial production chains, where each link can be controlled, is an effective way to reduce pollution. Impact assessments of chemicals are often hampered by missing data, and process control methods are difficult to use. For this reason, we need complete data inventories and improve data collection for chemical footprints, and companies should establish process inventories for the production of their products. On this basis, countries (regions) should work together to promulgate relevant laws and regulations, standardize building databases and promulgate protocols for confidentiality and sharing, interface, restrictions, and priority use of databases.
4. Study of a chemical-based safeguard mechanism for environmental management. For policymakers, an effective early warning system for potential environmental problems is necessary. This need is reflected by the need for policy development focusing on prevention at the source [58]. Media management based on the pollution control of water, soil, and air cannot effectively solve the problems of environmental pollution caused by the use of chemicals and the cumulative environmental pollution caused by chemical emissions. For environmental governance, the formulation of laws and regulations should be strengthened to enhance the operability and institutional support for the whole life cycle management of chemicals. The objectives, scope, standards, operational means, and regulations for the whole life cycle management of chemicals should be clarified and standardized.

## Figures and Tables

**Table 1:** List of key results and characteristic classification of product chemical footprint.

Type of Evaluation	Scholar	Research Object		Accounting Boundary		Data Sources	Calculation Method	Objective	Result
		Products	Kinds	Temporal Boundary	Spatial Boundary				
process evaluation	Qian J.H. et al. [16]	denim	industry	“cradle-to-gate”	Inputs of chemicals and releases of chemical pollutants in the chain of processes to be accounted for	USEtox2.01 database; Roos et al.	USEtox	Quantitative evaluation of toxicity in the production of textile and garment products	The chemical footprint of the warp process is higher than that of the weft process; among the chemical pollutants emitted, the reaction products of dimethyl (siloxane and polysiloxane) and silica have the greatest impact on human toxicity, and methylisothiazolinone has the greatest impact on ecotoxicity; the main sources of chemical pollutants are defoamers and detergents
process evaluation	Berthoud et al. [17]	French winter wheat	agriculture	cultivation, sowing, fertilizer and pesticide application, harvest, grain storage	On-farm fuel consumption for wheat production and harvesting	French Union of Agricultural Cooperatives (Invivo) and its decision-making tools for collecting data on agricultural lands, ecoinvent database	USEtox, LCA	Assessment of environmental impacts of winter wheat using LCA; assessment of partial freshwater ecotoxic effects due to pesticide use; identification of alternative active ingredients for highly effective pesticides	When considering the impact of freshwater ecotoxicity, pesticide use dominates the entire life cycle of winter wheat; the fertilization process has the greatest toxic impact; the observed results have significant scatter between fields compared to the low scatter between the four production scenarios; replacing the active ingredient with the greatest impact reduces the average impact
process evaluation	Li Y.et al. [18]	a polyester dress	industry	pre-treatment, dyeing and/or printing, finishing (“gate-to-gate”)	Raw materials for dyes, auxiliaries, and other chemicals	Swedish Foundation for Future Fashion Research	USEtox,LCIA	Analysis and identification of priority ecotoxic footprint control chemicals and priority ecotoxic footprint control processes for three dyeing and printing processes.	Among the three processes of pretreatment, dyeing and printing, the printing process has the largest ecotoxic footprint, and the end products of dyestuffs, thickeners, fluorescent brighteners and reduction inhibitors are the chemicals with the largest ecotoxic footprint.
end evaluation	Roos et al. (2015) [19]	hospital garments	industry	“gate-to-tomb” “gate-to-gate”	Dyestuffs, liquor ratio, auxiliary chemicals, energy sources	SC suppliers ecoinvent database	LCA, The Score System	Benefits and challenges of LCA, unbleached versus bleached garments toxicity comparison	LCA adds value to chemical performance assessment of textiles; potential for toxic effects of textile chemicals to affect the environmental performance ranking of textile products issues
end evaluation	Van Hoof et al. [20]	laundry products	industry	“cradle-to-grave”	Chemical inputs in the time boundary	Usetox database, Detergent Ingredient Database List	USEtox and critical dilution volume approaches, LCA	The toxicological effects of two detergents released into the environment were quantified and the advantages and disadvantages of each were compared using CDV and USEtox to account for more than 60 chemical components of the released pollutants.	The structural mechanisms of the model are well suited to simulate and explain the processes by which chemicals enter the environment and produce toxicity; in the CDV method, the dilute form has the greatest impact on ecotoxicity, while in the USEtox method, the powder form has the greatest impact.
end evaluation	García et al. [21]	pharmaceutical and personal care products	industry	“gate-to-tomb”	Chemicals contained in pharmaceutical and personal care products (PPCPs)	Experimental data, identification of databases, estimation using EPI suite and USEtox	LCA, USEtox, impact score (IS)	Characterization factors for 27 PPCPs estimated to be widely used worldwide, with a Spanish toxicity impact score classification for 49 PPCPSs	Pollutants discharged to continental freshwater tanks show the highest CFs values for human impact; freshwater aquatic ecotoxicity CFs are significantly higher than human toxicity CFs; the most impactful PPCPs according to the Spanish toxicity impact score are hormones, antidepressants, perfumes, antibiotics, angiotensin receptor blockers and lipids
end evaluation	Elorri Igos et al. [22]	dishwasher detergents	industry	partial treatment in a wastewater treatment plant (WWTP)	the dishwasher effluent composition after partial treatment in WWTP	European REACH frammework, the EPI Suite™ model, the European Chemicals Agency portal, eChem Portal, PAN Pesticide Database, ChemIDplus	USEtox, LCA	Quantifying the human toxicity and ecotoxicological effects of changes in detergent composition (phosphate/phosphate-free/ecologically labelled).	The freshwater ecotoxicity of dishwasher effluent composition can be more than 95 per cent, while the percentage of human toxicity is less than 36 per cent. The main contributors to freshwater ecotoxicity are sodium percarbonate and sodium triphosphate, with zinc making the largest contribution to human toxicity.
Source evaluation	Li Y et al. [23]	jeans	clothing/textile	The time span from the start of the wet treatment (i.e., washing) to the end of the wet treatment (i.e., washing).	The inputs and outputs of the various chemical substances involved in the time boundary.	Public report of the Mistra Future of Fashion research project in Sweden; CAS database	USEtox	Explore the ChF approach to textile and apparel products.	Logarithmic plots and cluster analysis indicated that the reaction products of dimethylsiloxane and silica and 2-methyl-4-isothiazoline-3-1 were the main sources of human and ecotoxicity, respectively, during the warp dyeing stage. In the weft bleaching stage, MgCl2 is highly toxic to humans and sulfuric and nonylphenol ethoxylates are highly toxic to the ecology.

## Data Availability

The study did not report any data.
